# Nickel removal by biosorption onto medlar male flowers coupled with photocatalysis on the spinel ZnMn_2_O_4_

**DOI:** 10.1186/2052-336X-12-13

**Published:** 2014-01-08

**Authors:** Ahmed Chergui, Farid Madjene, Mohamed Trari, Ali Khouider

**Affiliations:** 1 Laboratory of Electrochemistry-Corrosion, Metallurgy and Inorganic Chemistry, Faculty of Chemistry, Algiers, Algeria; 2 Laboratory of Storage and Valorization of Renewable Energies, Faculty of Chemistry, Algiers, Algeria

**Keywords:** Biosorption, Nickel, Medlar male flower, Photocatalytic

## Abstract

Ni^2+^ is a highly toxic above 0.07 mg/L and its removal is of high significance. The biosorption of Ni^2+^ onto medlar male flowers (MMF) was studied in relation with the physical parameters like pH, contact time, biosorbent dosage, Ni^2+^ concentration and temperature. The interaction biosorbent-Ni^2+^ was examined by the FTIR technique. The equilibrium was achieved within 40 min and the data were well fitted by the Langmuir and Redlich-Peterson (R-P) models. The maximum Ni^2+^ uptake capacity was 17.073 mg/g at 25°C and the Ni^2+^ removal follows a pseudo-second order kinetic with activation energy of 13.3 kJ/mol. The thermodynamic parameters: ΔS°, ΔH° and ΔG° showed that the biosorption was spontaneous and endothermic. MMF was used as a post treatment technique and the biosorption was coupled with the visible light driven Ni^2+^ reduction over the spinel ZnMn_2_O_4_. The effect of the pH, ZnMn_2_O_4_ loading and light intensity on the photoactivity was investigated. 77.5% of Ni^2+^ was reduced after ~140 min under optimal conditions. The Ni^2+^ removal reached a rate conversion of 96% of with the coupled system biosorption/photocatalysis is very promising for the water treatment.

## Introduction

Extensive industrial activities have led to tremendous increase in the use of toxic metals over the last decades provoking large scale pollution. Soil and water were continuously contaminated by heavy metals coming from various industries. Ni^2+^ cannot be biodegraded unlike organic pollutants; it persists indefinitely and accumulates through the food chain, thus posing a serious threat to human health [1]. Nickel is known as one of the most common toxic metals and inhaled nickel compounds are carcinogenic and provoke asthma, chronic bronchitis, pulmonary embolism as well as respiratory and may bring nausea, dizziness and diarrhea [2,3]. The WHO has drastically reduced at 0.07 mg/L the authorized threshold for nickel in drinking water [4]. Therefore, it was necessary to treat metal-contaminated water prior its discharge in the aquatic environment. Several techniques have been used to this end, like precipitation, ion exchange, biosorption and membranes [5]. In recent years, considerable attention has been focused on the biosorption using agricultural materials [6,7]. Hence, efforts were done to develop inexpensive biosorbents using agricultural wastes. We have tested several biological materials for the metals removal and studies were carried out to investigate the potential of locally available biosorbent namely the medlar male flowers (MMF) for the nickel removal from aqueous solutions. The contact time, biosorbent dose, pH, Ni^2+^ concentration and temperature were optimized. To understand the nature of the Ni^2+^ biosorption on MMF, equilibrium isotherms were analyzed by the Freundlich, Langmuir and Redlich-Peterson (R-P) models. Kinetic and thermodynamic parameters were also evaluated.

On the other hand, the biosorption greatly reduces the pollution but often not enough to comply with the standards of the world health organization. At this level, the photocatalysis appears to be quite promising because of its simplicity. There were only few studies on the combined systems (biosorption/photocatalysis) for the water treatment. So, in a second step, the system was directed to the spinel photocatalyst ZnMn_2_O_4_ which was presented as having the required photoelectrochemical parameters. Its conduction band (CB) derives from *3d* orbital able to reduce metal ions to the elemental states. In addition, it is low cost, non-toxic and chemically stable under the operating conditions.

### Experimental

MMF obtained from local farms (Algiers) is thoroughly washed with water and dried at 80°C. Then, it was crushed, ground in an agate mortar and sieved to select a particle size of 500 μm by using an ASTM standard sieve. It was stored in closed bottles until use.

**Figure 1:**
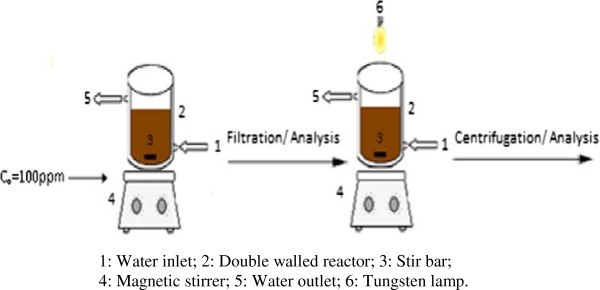
A schematic diagram of the coupled biosorption/photocatalysis system.

The Ni^2+^ stock solution (1 g/L) was prepared by dissolving the accurate quantity of NiSO_4_, 6H_2_O (Merck, 99%) in distilled water. Concentrations down to 50 mg/L were prepared by dilution. The pH was adjusted to the desired value by addition HCl or NaOH using a digital pH meter (Schott 825). The batch experiments were done in a double walled Pyrex reactor of 500 cm^3^ capacity (Figure 1) whose temperature was regulated by a thermostated bath (Julabo) under magnetic stirring (500 rpm). This was achieved to investigate the optimal conditions of pH (2–7), biosorbent dosage (3–9 g/L), Ni^2+^ concentrations (50–250 mg/L) and temperature (15–55°C). The aliquots were withdrawn at regular time intervals and filtered off. The supernatant was analyzed for residual Ni^2+^ concentration with a double beam UV-Visible spectrophotometer (Shimadzu 1800). The solution was mixed to dimethylglyoxime in presence of bromine in ammoniacal medium and the complex was titrated at 465 nm. The turbidity was measured using an Aqualytic turbidimeter (WTW Turb 550). The amount of Ni^2+^ uptake and the biosorption percentage were calculated from the relations:

(1) $$q=\left(C_{o}-C\right)\frac{V}{m_{s}}$$

(2) $$\;\left(\%\right)=\frac{C_{o}-C}{C_{o}}\times 100$$

The FTIR spectra of unloaded and Ni^2+^ loaded MMF were recorded on an infrared spectrometer (Jasco-3200) using the KBr routine technique. The specific surface area was measured by the BET method with a Quadrasorb SI apparatus (Micromeritics ASAP 2010). The point of zero charge (pzc) was determined by the immersion technique described elsewhere [8].

The spinel ZnMn_2_O_4_ was synthesized by sol–gel. A stochiometric mixture of Zn(NO_3_)_2_, 6H_2_O/Mn(NO_3_)_2_, 6H_2_O (0.025 M/0.05 M), both of purity greater than 99%, was dissolved in 60 mL of ethylene glycol at room temperature. The solution was heated under stirring at 70°C (6 h) under reflux and the viscous solution was dried at 120°C. The amorphous powder was heated at 850°C and furnace cooled. The phase was confirmed by X-ray diffraction (XRD) using monochromatized Cu Kα radiation. The photocatalytic tests were carried out in the same reactor (Figure 1). The light source was a 200 W tungsten lamp (Osram) disposed at 10 cm above the reactor. The light flux was measured with a commercial light meter (roline RO 1332). The light was turned on after a transition period of 2 h for the dark adsorption onto the spinel.

## Results and discussion

### Characterization of the biosorbent

**Figure 2:**
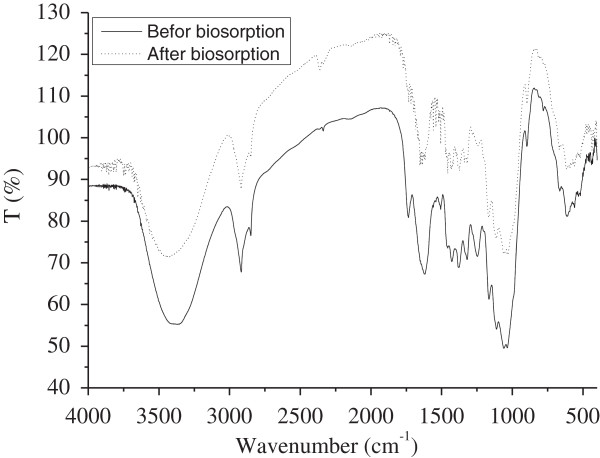
**FTIR spectra of (a) unloaded and (b) Ni**^**2+ **^**loaded MMF. g/L).**

The physical characteristics of MMF were summarized in Table 1. The FTIR spectra of unloaded and Ni^2+^-loaded MMF (Figure 2) were compared to get insights on the nature of the biosorbent-Ni^2+^ interactions and to determine the frequency changes in the functional groups for Ni^2+^ binding. The spectrum of MMF shows a strong band at 3444 cm^-1^ attributed to hydroxyl groups. The peak at 2920 cm^-1^ is due to the C–H stretching frequency whereas that at 1644 cm^-1^ is assigned to C = O stretching of the primary and secondary amides (NH_2_CO). The peaks at 1505 and 1382 cm^-1^ are due to N–H stretching of primary and secondary amides, and amide (III) respectively. Bands at 1320 and 1243 cm^-1^ are characteristic of carboxylic acids. The strong C–O band at 1057 cm^-1^ confirms the lignin structure of MMF [9]. The FTIR spectrum of Ni^2+^ loaded MMF (Figure 2b) shows a decrease in intensity and a shift in asymmetric stretching frequencies at 3444, 1644 and 1505 cm^-1^ due to coordinated Ni^2+^ ions by hydroxyl, carboxyl, and amides groups respectively on the MMF surface.

**Table 1:** Physico-chemical characterization of the MMF

**Parameters**	
**Particle size (mm)**	**0.5**
**BET surface area (m**^**2**^**/g)**	**2.78**
**pH**_**PZC**_	**7.26**

### Effect of contact time

**Figure 3:**
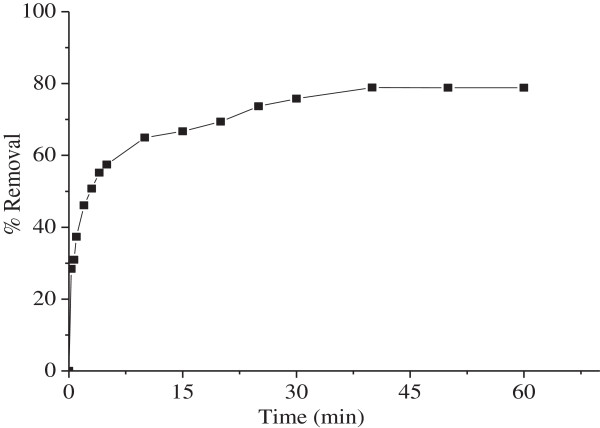
**Effect of contact time for biosorption of Ni**^**2+ **^**onto MMF (25°C, C**_**o**_ **= 100 mg/L, pH = 5.5, m = 5.**

The effect of the contact time on the rate of Ni^2+^ removal is shown in Figure 3. The process was rapid at the beginning due to large surface area of the biosorbent and gradually decreases to reach an equilibrium state. The plots reveal that the maximum percentage of Ni^2+^ removal occurs after 40 min of shaking. Then, the biosorption becomes controlled by the rate of Ni^2+^ transported to internal sites of the grains.

### Effect of the biosorbent dose

**Figure 4:**
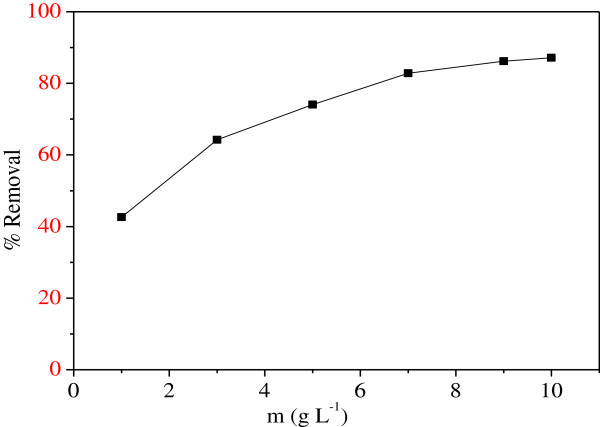
**Effect of biosorbent dose for Ni**^**2+ **^**biosorption onto MMF (25°C, t = 40 min, C**_**o**_ **= 100 mg/L, pH = 5.5).**

As expected, the Ni^2+^ biosorption increases with increasing the MMF dose, and becomes almost constant beyond 7 g/L (Figure 4). With increasing the MMF dose, more surface area is available due to increasing number of active sites, thus making easier the penetration of Ni^2+^ to the biosorption sites. So, we have used a biosorbent dose of 7 g/L for the following experiments. Further increase of the mass does not produce a significant effect on the Ni^2+^ biosorption because of the saturation effect. This new biosorbent could not only remove Ni^2+^, but also produces a weak turbidity not exceeding 1.73 NTU which an acceptable value. It is worthwhile to outline that a low turbidity is an important requirement for the water treatment.

**Figure 5:**
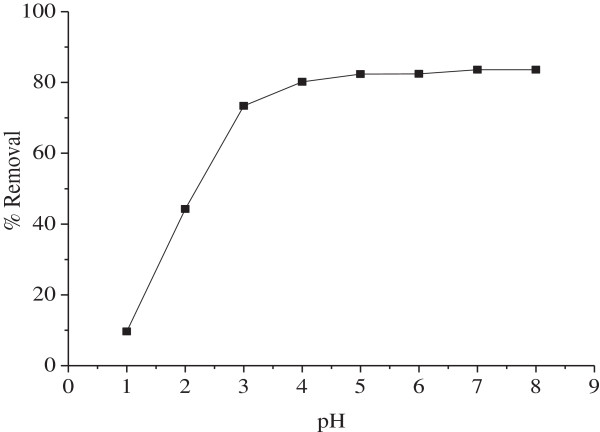
**Effect of pH for Ni**^**2+ **^**biosorption onto MMF (25°C, t = 40 min, C**_**o**_ **= 100 mg/L, m = 7 g/L).**

The pH is an important parameter for controlling the biosorption and its influence on the Ni^2+^ uptake by MMF is illustrated in Figure 5. Experiments were done by varying the pH over the range (2–7) at 25°C with MMF dose of 7 g/L. The performance increases rapidly with increasing pH up to 5, then slowly and beyond pH 8.5, Ni(OH)_2_ precipitates owing to the low solubility product (~10^-16^). At acidic pHs, the MMF surface is covered by H^+^ ions and Ni^2+^ cannot compete for biosorption sites. The increase in the biosorption at higher pHs is likely associated with deprotonation of the biosorbent surface or other negatively charged groups lead to electrostatic attraction of Ni^2+^ ions. Based on these results, the best biosorption occurs at pH ~ 7 with a percentage removal of 83%. Accordingly, the further biosorption studies are carried around this pH which is close to the real pH of naturel medium.

### Effect of temperature

**Figure 6:**
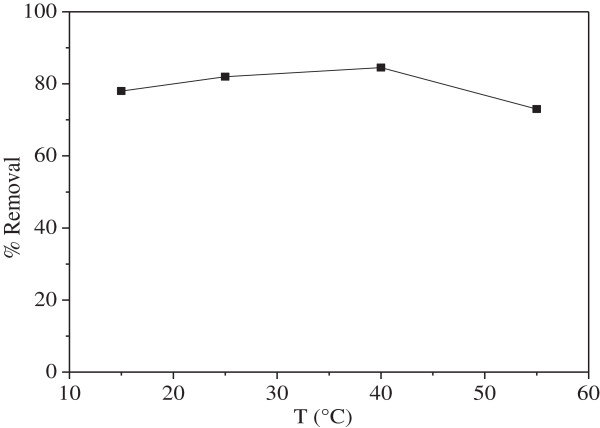
**Effect of temperature for Ni**^**2+ **^**biosorption onto MMF (C**_**o**_ **= 100 mg/L, t = 40 min, pH = 5.5, m = 7 g/L).**

The thermal variation of the Ni^2+^ uptake at a concentration of 100 mg/L onto MMF is shown in Figure 6. An increase of the temperature results in an enhanced biosorption rate and the maximum uptake occurs at 40°C. At that temperature, an increasing number of molecules acquire sufficient energy to interact with active sites. Furthermore, raising temperature produces a swelling effect within the internal MMF structure, thus enabling large amount of Ni^2+^ ions to access to inside cavities.

The Ni^2+^ removal, evaluated for the biosorption isotherms, clearly indicates an endothermic process. However, the biosorption was found to decrease at higher temperatures (55°C) possibly due to the damage of active binding sites in the biomass. In addition, the water vaporization becomes an increasing problem. Nevertheless, the temperature-dependence is somewhat different in our case, and this may be due to the differences in the experimental conditions and the nature of the biosorbent. As a result, the temperature of 40°C is found to be optimal. However, the Ni^2+^ biosorption at 40°C (84%) is close to that at 25°C (82%). So, to save energy the last temperature is selected for further experiments.

### Effect of initial concentration

**Figure 7:**
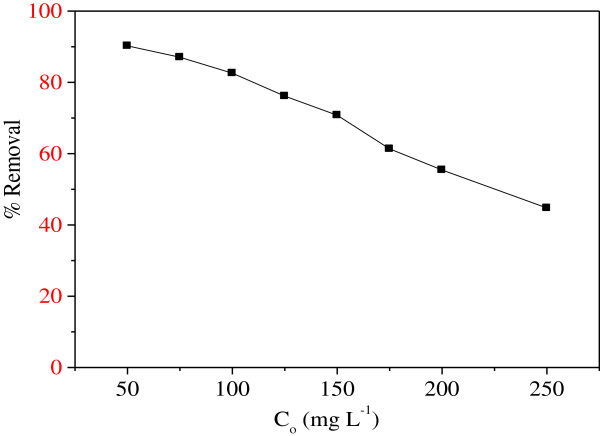
**Effect of Ni**^**2+ **^**concentration for biosorption onto MMF (25°C, t = 40 min, pH = 5.5, m = 7 g/L).**

Nickel comes from industrial activities and discharged in the aquatic medium at concentration up to 400 mg/L. So it is interesting to study the effect of concentration. The equilibrium Ni^2+^ uptake for different concentrations (50–250 mg/L) at 25°C on MMF (7 g/L) at pH ~ 7 is shown in Figure 7. The biosorption rate decreases with increasing Ni^2+^ concentration from 83% (50 mg/L) to 62% (250 mg/L). At low Ni^2+^ concentrations, the binding sites are unsaturated and consequently available for the biosorption process. By contrast, for high Ni^2+^ concentrations the number of ions competing for the available sites on the biomass increases, thus resulting in a decrease of the Ni^2+^ biosorption.

### Biosorption isotherms

The analysis of the isotherm by fitting the experimental data to various models is an important step for the design purpose. Three models are tested namely the Langmuir, Freundlich and Redlich–Peterson (R-P) ones. a) The Langmuir model is successfully used for homogenous biosorption surface [2]:

(3) $$q_{e}=\frac{q_{m}\;b\;C_{e}}{1+b\;C_{e}}$$

**Figure 8:**
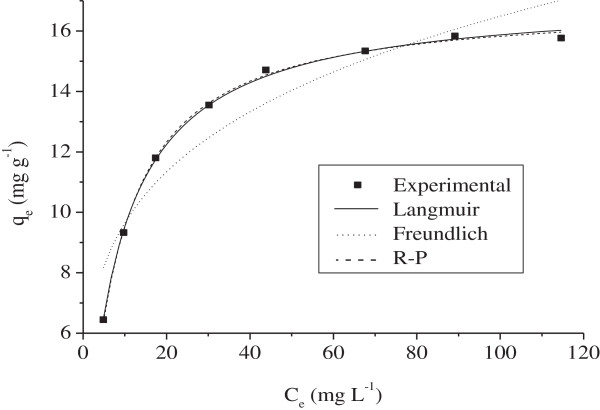
**Isotherms of Ni**^**2+ **^**biosorption onto MMF adjusted to the Langmuir, Freundlich and R-P models (25°C, t = 40 mn, pH = 5.5, m = 7 g/L).**

Figure 8 illustrates the plots at 25°C, the constants q_m_ and b are gathered in Table 2.

**Table 2:** **The isotherm constants for the Ni**^**2+**^**biosorption onto MMF under optimized conditions for Langmuir Freundlich and Redlich-Peterson models**

**Langmuir**			**Freundlich**			**Redlich-Peterson**			
**q**_**m**_	**b**	**R**^**2**^	**N**	**K**_**f**_	**R**^**2**^	**K**_**R**_	**a**_**R**_	**g**	**R**^**2**^
**17.143**	**0.125**	**0.9984**	**0.232**	**5.650**	**0.9054**	**1.996**	**0.104**	**1.024**	**0.9990**

The high coefficients R^2^ show that the Ni^2+^ biosorption onto MMF follows the Langmuir model whose main feature is expressed in term of a dimensionless separation constant (R_L_) [10]:

(4) $$R_{L}=\frac{1}{1+b\;C_{o}}$$

The R_L_ value indicates the type of isotherm: unfavorable (R_L_ > 1), linear (R_L_ = 1), favorable (0 < R_L_ < 1) or irreversible (R_L_ = 0) [10]. The R_L_ values for different Ni^2+^ concentrations are given in Table 3. It can be observed that R_L_ lies in the range 0–1 in all experiments, thus confirming the favorable Ni^2+^ uptake onto MMF.

**Table 3:** **The separation factor versus the initial Ni**^**2+**^**concentration**

**C**_**o **_**(mg/L)**	**40**	**60**	**80**	**100**	**150**	**200**	**250**
**R**_**L**_	**0.0868**	**0.0596**	**0.0454**	**0.0366**	**0.0247**	**0.0186**	**0.0150**

The maximum biosorption capacity (q_m_) is compared with other biosorbents reported in the literature (Table 4), where q_m_ has not been derived specifically. One can see that the maximum Ni^2+^ biosorption capacity is intermediate compared to other biosorbents and MMF is a promising for the water treatment. b) The Freundlich model is suitable for heterogeneous surfaces [11]:

(5) $$q_{e}=K_{f}\;C_{e}^{1/n}$$

**Table 4:** **Biosorption capacities of different biosorbents for Ni**^**2+**^**removal from water**

**Biosorbent**	**q**_**m **_**(mg/g)**	**Ref.**
**Gracilaria caudata**	**45**	[2]
**Sargassum muticum**	**70**	[2]
**Tea factory waste**	**15.2**	[3]
**Activated sludge**	**238.1**	[5]
**Baker’s yeast**	**8.2**	[12]
**Aspergillus niger**	**4.82**	[13]
**Medlar male flower**	**17**	**This study**

Figure 8 shows the plots while the constants K_f_ and 1/n are given in Table 2. The values of 1/n, less than 1, indicating a favorable biosorption. However, the theoretical values deviate from the experimental data suggesting that the model is not appropriate to describe the Ni^2+^ biosorption onto MMF.

c) The R–P model combines both the Langmuir and Freundlich ones and the hybrid mechanism does not follow a monolayer biosorption [14]:

(6) $$q_{e}=\frac{K_{R}\;C_{e}}{1+a_{R}C_{e}^{g}}$$

The exponent g varies between 0 and 1. It is helpful to outline that for g = 1, the equation converts simply to the Langmuir isotherm while for g = 0, it is simplified to the Henry’s law. For $1<<a_{R}.C_{e}^{g}$, the model is identical to the Freundlich one. Table 2 lists the biosorption parameters together with the R^2^ values. A comparison between the theoretical and experimental data of Ni^2+^ biosorption onto MMF was well illustrated in Figure 8. The R–P equation applied over the whole concentrations range gives a coefficient R^2^ close to 1, thus confirming the best fit for Ni^2+^ biosorption for the two models. The g values were close to unity and this means that the isotherm approaches the Langmuir model rather than the Freundlich one. Hence, the good fit of the equilibrium data for Langmuir and R–P isotherms confirms the monolayer coverage of Ni^2+^ onto MMF.

### Biosorption kinetic

**Figure 9:**
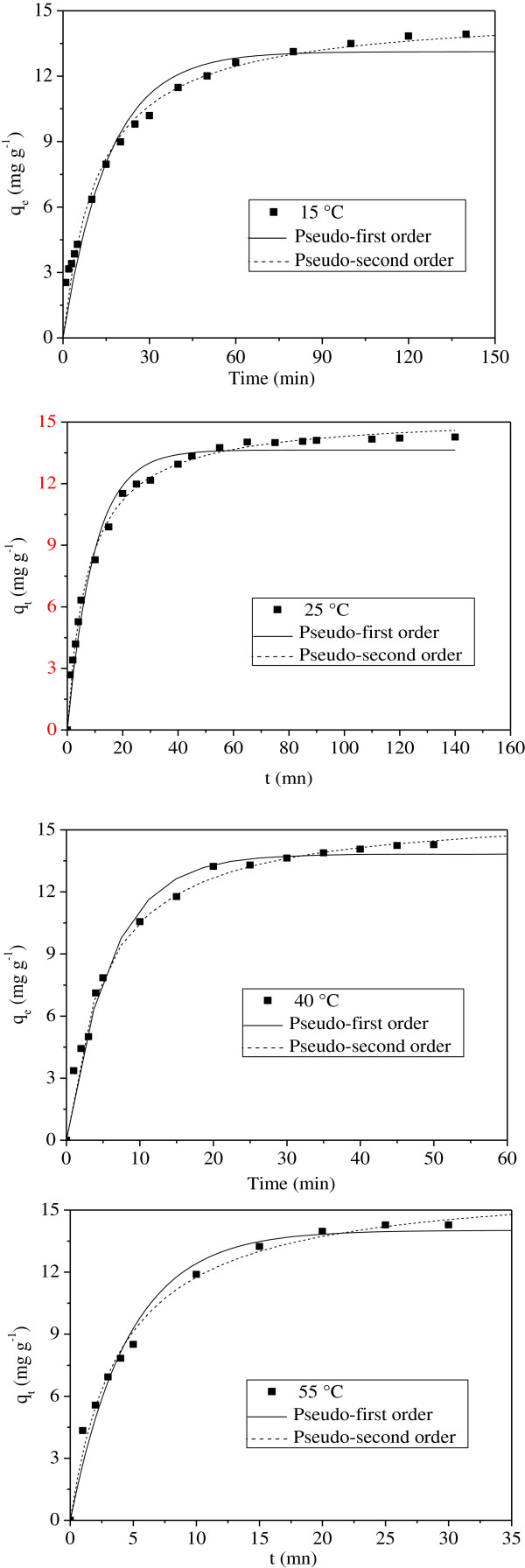
**Biosorption kinetics plot of Ni**^**2+ **^**onto MMF biomass.**

The biosorption kinetic is important from a practical point of view since it controls the efficiency of the process and the models correlate the Ni^2+^ uptake rate with the bulk concentration. In order to analyze the biosorption rate, the kinetic data were modeled with the Lagergren pseudo-first-order and pseudo-second order equations [15].

(7) $$\mathrm{Pseudo}\;-\;\mathrm{first}\;-\;\mathrm{order}:\;q_{t}=q_{e}\left(1-e^{-k_{1}t}\right)$$

(8) $$\mathrm{Pseudo}\;-\;\mathrm{second}\;-\;\mathrm{order}:\;q_{t}=q_{e}\frac{q_{e}k_{2}t}{1+q_{e}k_{2}t}$$

where q_t_ and q_e_ are the amounts of Ni^2+^ adsorbed (mg/g) at time (t) and at equilibrium, respectively, k_1_ (min^-1^) and k_2_ (mg/g min) are the corresponding rate constants. q_t_, k_1_, and k_2_ were calculated by the non-linear regression analysis (Figure 9) using the Origin program (version 7.5). The optimization uses the error function to fit the experimental data.

The coefficients R^2^ and the chi-square test (χ^2^) were used to measure the goodness-of-fit:

(9) $$\chi^{2}=\sum_{i=1}^{N}\frac{\left(q_{e}-q_{i}\right)}{q_{i}}$$

where q_e_ is the observation from the batch experiment, q_i_ the estimate from the isotherm for corresponding q_e_ and N the number of observations in the experimental isotherm. If the data from the model are similar to the experimental ones, χ^2^ is small [16]. The coefficients R^2^ and χ^2^ in the temperature range (15–55°C) indicate that the biosorption kinetic was well described by the pseudo-second-order equation (Table 5). The comparison of the experimental and theoretical values of q_t_ evaluated from Eq. (8) shows that for all temperatures, q_t_ is almost constant and increases with temperature along with k_2_.

**Table 5:** **Kinetic parameters for Ni**^**2+**^**biosorption onto MMF at different temperatures**

**T (K)**	**Pseudo-first-order**			**Pseudo-second-order**		
	**k**_**1**_	**χ**^**2**^	**R**^**2**^	**k**_**2**_	**χ**^**2**^ **× 10**^**3**^	**R**^**2**^
	**(min**^**-1**^**)**			**(g/mg min)**			
**288**	**0.0634**	**0.2113**	**0.9599**	**0.1327**	**3.056**	**0.9880**
**298**	**0.1006**	**0.2711**	**0.9425**	**0.1536**	**0.566**	**0.9863**
**313**	**0.1641**	**0.1769**	**0.9315**	**0.2499**	**0.762**	**0.9800**
**328**	**0.2169**	**0.8849**	**0.9770**	**0.2987**	**0.025**	**0.9938**

### Thermodynamic parameters of biosorption

The free energy (ΔG°) of the biosorption is related to the equilibrium constant (k_c_) by [3]:

(10) $$\mathrm{\Delta G}=\;-\mathrm{RT}\ln k_{c}=\mathrm{\Delta H}-\mathrm{T\Delta S},\;k_{c}=\frac{C_{o}-C_{e}}{C_{e}}$$

The standard enthalpy (ΔH°) and entropy (ΔS°) were obtained from the linear plot lnk_c_ versus 1/T. The negative value of ΔG° (Table 6) indicates a spontaneous biosorption and the low temperatures make the biosorption easier. The positive values of ΔH° and ΔS° implies that the Ni^2+^ biosorption onto MMF was endothermic with increased randomness at the solid/solution interface.

**Table 6:** **Thermodynamic parameters for the biosorption of Ni**^**2+**^**onto MMF at different temperatures**

**T (K)**	**ΔG°**	**ΔH°**	**ΔS°**
	**(kJ/mol)**	**(kJ/mol)**	**(J/mol K)**
**288**	**-3.460**		
**298**	**-3.921**	**9.806**	**46.065**
**313**	**-4.612**		
**328**	**-5.303**		

### Photocatalytic process

As mentioned above, the Ni^2+^ uptake on MMF greatly reduces the pollution but not sufficiently to comply with the standards of the water quality. However, the biosorption can be used as post treatment for the photocatalyic process. Using colloidal semiconductor particles as light absorbing units is simple and does not need any sophisticated device. The first step was to generate electron/hole (e^-^/h^+^) pairs on a semiconductor by energetic photons (hν > Eg). Ni^2+^ was photo-electrochemically reduced to elemental state. In this respect, the spinel ZnMn_2_O_4_ shows light absorption for wavelengths shorter than 730 nm. In addition, the oxide is chemically stable and has been elaborated via sol–gel route in order to increase the active surface.

**Figure 10:**
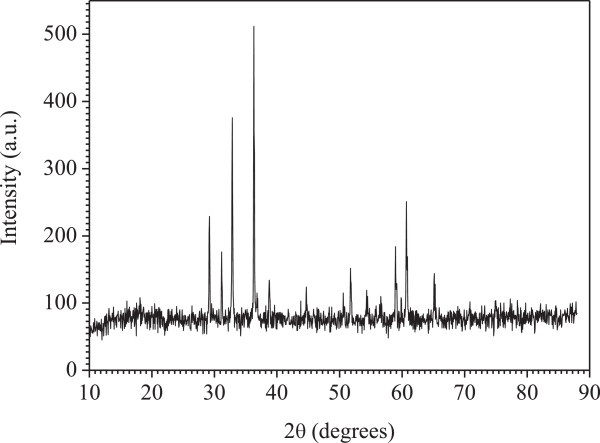
**XRD pattern of ZnMn**_**2**_**O**_**4 **_**synthesized by sol–gel route.**

Even though the photocatalytic processes are related to the morphology of the material, a detailed understanding of the fundamental properties is required to get a high-quality powder. ZnMn_2_O_4_ was synthesized by sol–gel and the XRD pattern (Figure 10) is characteristic of single phase. All peaks are assigned to the spinel phase in agreement with the JCPDS card N° 24–1133. Nevertheless, when the calcination temperature exceeds 850°C, the crystallites can agglomerate in grains and decrease the specific surface area. This has been corroborated by the lack of XRD peak broadening and the relatively small active surface (28 m^2^/g).

**Figure 11:**
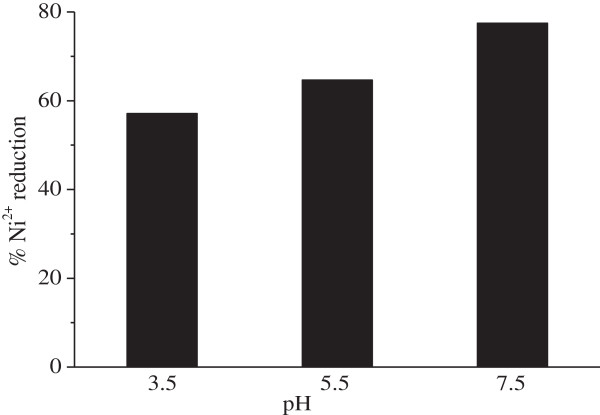
**Influence of pH on the Ni**^**2+ **^**photoreduction.** (25°C, C_o_ = 18 mg/L, m_s_ = 0.5 g/L, flux = 10.7 mW/cm).

The main interest of this section was to investigate the effect of some physical parameters on the Ni^2+^photoreduction. A transition period is required before irradiation and the suspension ZnMn_2_O_4_ was maintained in the dark during this period. The starting concentration was set at 18 ppm and the Ni^2+^ solution was equilibrated with the catalyst during 2 h to establish the adsorption conditions. The difference between the nominal concentration and that of measured after equilibrium was taken as the quantity of adsorbed Ni^2+^ onto ZnMn_2_O_4_ (∼5%). The pH of the solution has a direct influence on the photo-electrodeposition via the surface charge catalyst. With Ni^2+^, the adsorption follows a cationic type law and is pronounced at high pH. We have conducted the experiments for 140 min at different pHs and the corresponding fractional reduction of Ni^2+^ is presented in Figure 11. It appears that the photoreduction was maximum (~77.5%) at pH 7.5 and decreased rapidly with decreasing pH, in acidic media, the Ni^2+^ were repelled by the positive surface charge of ZnMn_2_O_4_. In the pH range (3.5-7.5) the photocatalytic Ni^2+^ reduction on ZnMn_2_O_4_ was rather limited.

**Figure 12:**
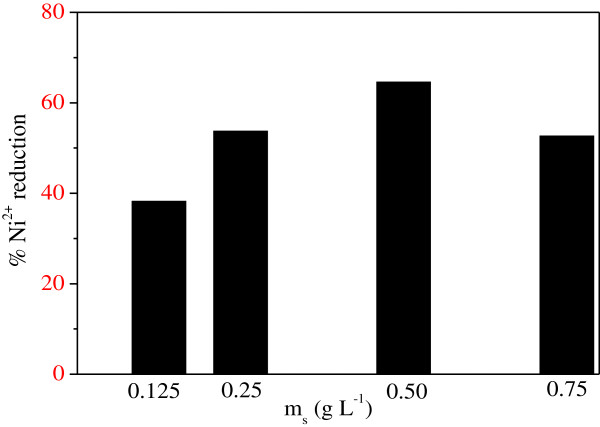
**Influence of the mass of ZnMn**_**2**_**O**_**4 **_**on the Ni**^**2+ **^**photoreduction (25°C, C**_**o**_ **= 18 mg/L, pH = 7.5, light flux = 10.7 mW/cm).**

The photocatalytic tests were carried out with various amounts of ZnMn_2_O_4_ and the rate of Ni^2+^ reduction increases with increasing the mass of ZnMn_2_O_4_ (Figure 12). For smaller doses, less actives sites for the reduction process are available and the photoactivity increases in parallel with the amount of ZnMn_2_O_4_. On the contrary, for higher doses, all photocatalytic sites are saturated and the light scattering of the incident flux, the shadowing effect of the powder catalyst; the metal clusters account for the decrease of the activity.

**Figure 13:**
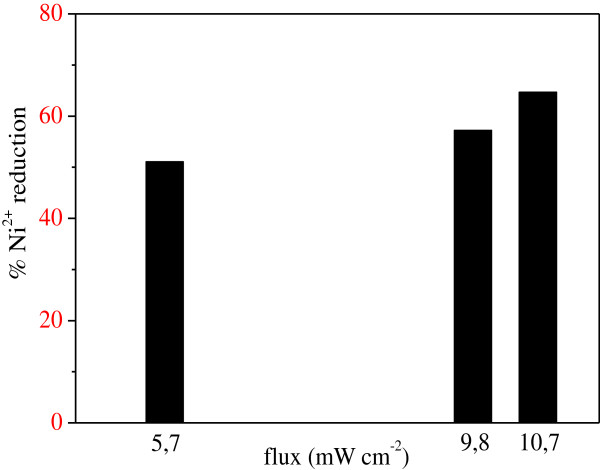
**Influence of the light intensity on the Ni**^**2+ **^**photoreduction (T = 25°C, C**_**o**_ **= 18 mg/L, m**_**s**_ **= 0.5 g/L, pH = 7.5).**

Indeed, the incident light on the metal surface was partially absorbed and converted to heat. The two opposing effects gave an optimal ZnMn_2_O_4_ dose of 0.5 g/L. Increasing light intensity enhanced both the rate and the extent of Ni^2+^ reduction (Figure 13).

Taking into account the adsorption and the photoreduction, the modified Langmuir–Hinshelwood model was successfully applied to assess the Ni^2+^ photoreduction rate [17].

(11) $$\frac{-\mathrm{dC}}{\mathrm{dt}}=k_{\mathrm{app}}C_{o}$$

**Figure 14:**
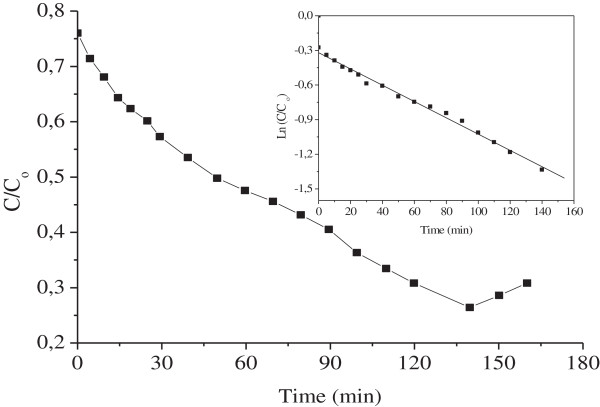
**Photoreduction of Ni**^**2+ **^**on ZnMn**_**2**_**O**_**4**_**.** Inset: The plot of Ln C/C_o_ vs. time.

The reaction occurs between adsorbed species on the spinel surface. The linear plot of lnC/C_o_ vs time indicates that the reduction obeys to a first order kinetic (Figure 14). The apparent reaction constant (k_app_) averages 0.42 h^-1^ and the half life, i.e. the time needed for the concentration to fall to half of its initial value, was found to be concentration independent and averages 1.5 h.

## Conclusion

An agricultural by product namely the Medlar male flower, locally available has been successfully used for Ni^2+^ biosorption from aqueous solution. The contact time, biosorbent dose, pH and temperature were optimized. The maximum biosorption capacity was obtained at pH 5, and the equilibrium was reached in less than 40 min. The isotherms data were well fitted with both the Langmuir and Redlich-Peterson models. The negative free energy confirmed a favorable Ni^2+^ biosorption where the positive enthalpy indicated an endothermic nature. The spinel ZnMn_2_O_4_ elaborated by co-precipitation, was used to reduce the remaining concentration by photocatalysis into elemental state. A reduction rate of 77.5% was achieved after 2 h irradiation at pH 7.5 with an optimal ZnMn_2_O_4_ mass equal to 0.5 g/L. The photoreduction followed a first order kinetic. The results indicated that the coupled system may be a viable alternative and a new way for the Ni^2+^ removal in aquatic medium.

## Nomenclature

b Langmuir biosorption constant (L/mg)

C_e_ Residual Ni^2+^ concentration at equilibrium (mg/L)

C_o_ Initial Ni^2+^ concentration (mg/L)

Eg The optical gap

K_f_ The Freundlich constants denoting the biosorption capacity (mg^1-1/n^/g L^1/n^)

k_1_ Pseudo-first order biosorption rate constant (min^-1^)

k_2_ Pseudo-second order biosorption rate constant (g/mg min)

k_app_ The apparent reaction constant

k_c_ Standard thermodynamic equilibrium constant.

k_app_ The apparent reaction constant

m Biosorbent dosage (g)

m_s_ semiconducteur dosage (g)

n The Freundlich constants denoting the biosorption intensity

q_e_ Amount of Ni^2+^ adsorbed on the biosorbent at equilibrium (mg/g)

q_max_ Langmuir biosorption constant (mg/g)

q_t_ Amount of Ni^2+^ adsorbed on the sorbent at any time (mg/g)

R Universal gas constant

R^2^ Correlation coefficient

R_L_ Dimensionless separation factor

T Absolute temperature (K)

t Time of biosorption (min)

ΔG° Free energy (kJ/mol)

ΔH° Enthalpy change (kJ/mol)

ΔS° Entropy change (J/mol K)

## Competing interests

All authors declare that they have no competing interest.

## Authors’ contributions

AC and FM were the main authors, designed and performed the study and drafted the manuscript. MT supervised the work. AK helped in the biosorption study. All authors read and approved the final manuscript.
